# HIV-Associated Structural and Functional Brain Alterations in Homosexual Males

**DOI:** 10.3389/fneur.2021.757374

**Published:** 2022-01-14

**Authors:** Qiong Ma, Xiudong Shi, Guochao Chen, Fengxiang Song, Fengjun Liu, Huang Zheng, Yuxin Shi, Dan-Chao Cai

**Affiliations:** ^1^Shanghai Institute of Medical Imaging, Fudan University, Shanghai, China; ^2^Department of Radiology, Shanghai Public Health Clinical Center, Fudan University, Shanghai, China; ^3^Shanghai Commercial Sex Worker (CSW) & Man Have Sex With Man (MSM) Center, Shanghai, China

**Keywords:** homosexual, HIV infection, gray matter volume, functional connectivity, amplitude of low frequency fluctuation, regional homogeneity

## Abstract

**Purpose:**

Neuroimaging elucidations have shown structural and functional brain alterations in HIV-infected (HIV+) individuals when compared to HIV-negative (HIV–) controls. However, HIV− groups used in previous studies were not specifically considered for sexual orientation, which also affects the brain structures and functions. The current study aimed to characterize the brain alterations associated with HIV infection while controlling for sexual orientation.

**Methods:**

Forty-three HIV+ and 40 HIV– homosexual men (HoM) were recruited and underwent resting-state MRI scanning. Group differences in gray matter volume (GMV) were assessed using a voxel-based morphometry analysis. Brain regions with the altered GMV in the HIV+ HoM group were then taken as regions of interest in a seed-based analysis to identify altered functional connectivity. Furthermore, the amplitude of low-frequency fluctuation (ALFF) and regional homogeneity values were compared between the two groups to evaluate the HIV-associated functional abnormalities in local brain regions.

**Results:**

HIV+ HoM showed significantly increased GMV in the bilateral parahippocampal gyrus and amygdala, and decreased GMV in the right inferior cerebellum, compared with the HIV– HoM. The brain regions with increased GMV were hyper-connected with the left superior cerebellum, right lingual gyrus, and left precuneus in the HIV+ HoM. Moreover, the ALFF values of the right fusiform gyrus, and left parahippocampal gyrus were increased in the HIV+ HoM. The regional homogeneity values of the right anterior cingulate and paracingulate gyri, and left superior cerebellum were decreased in the HIV+ HoM.

**Conclusion:**

When the study population was restricted to HoM, HIV+ individuals exhibited structural alterations in the limbic system and cerebellum, and functional abnormalities in the limbic, cerebellum, and visual network. These findings complement the existing knowledge on the HIV-associated neurocognitive impairment from the previous neuroimaging studies by controlling for the potential confounding factor, sexual orientation. Future studies on brain alternations with the exclusion of related factors like sexual orientation are needed to understand the impact of HIV infection on neurocognitive function more accurately.

## Introduction

Even with the success of combination antiretroviral therapy (cART), various neurological complications caused by the infiltration of the human immunodeficiency virus (HIV) in the central nervous system (CNS) (1), especially HIV-associated neurocognitive disorder (2), remain a heavy disease burden. Neuroimaging is a vital tool to provide insight into structural, functional, and molecular changes occurring in the brain and has the potential to comprehensively elucidate the pathogenesis of HIV-associated neurocognitive disorder (3, 4). Structural magnetic resonance imaging (sMRI) studies of HIV-infected (HIV+) individuals have found widespread brain atrophy and volume reduction in the subcortical structures including caudate nucleus, putamen, amygdala, thalamus, hippocampus, and parahippocampus (5–8), and cerebellar (9, 10). Resting-state functional MRI (rs-fMRI) studies revealed that HIV+ individuals had certain attenuation in brain intra- and internetwork connections, especially in the default mode, salience, and executive control networks, which might underlie the reported cognitive impairments (11, 12). However, these studies were inconsistent in regional distribution and effect sizes of brain abnormalities, and their conclusions cannot be generalized to the whole HIV+ cohort.

The inconsistencies in neuroimaging studies of HIV infection might be caused by multiple confounding factors, including age, sex, and substance use. For instance, age and HIV synergistically deteriorate cognitive performance through overlapping pathogenic mechanisms (13). One study reported that age exacerbated HIV-associated white matter abnormalities and fronto-subcortical white matter integrity loss (14). Numerous neuroimaging studies also showed brain atrophy, white matter damage and functional network strength, as a result of substance use in persons living with HIV (4). Discrepant results from previous studies might also be due to the heterogeneity of clinical status in studied samples, such as cART initiation and adherence, route of HIV infection, stage of HIV infection, viral suppression, and CD4 cell counts. For example, HIV+ individuals with constant cART showed greater functional connectivity in fronto-striatal networks than HIV+ individuals without cART (15), which was possibly related to the reduced inflammatory response and glial activation during cART (16). To sum up, different confounding factors might have different effects on alterations in the structure and function of brain, which to some extent affects the understanding of HIV infection effects. And many other factors have not been taken into account.

Sexual orientation is a biological phenomenon for unknown reasons. Many previous studies have investigated the brain differences between differing sexual orientations after controlling for potential mediating factors including HIV infection and substance abuse. sMRI showed, compared with the heterosexual men (HeM), the homosexual men (HoM) displayed thinner visual cortex and thicker patietal cortex, smaller thalamus and larger corpus callosum (17–19, 48). A positron emission tomography study that measured the cerebral blood flow showed that the amygdala connections in HoM were more widespread from the left amygdala whereas those in HeM were more widespread from the right amygdala (20). Furthermore, a series of rs-fMRI studies revealed differences in regional homogeneity, amplitude of low-frequency fluctuation (ALFF), and functional connectivity between HoM and HeM (21, 22). Sexual transmission is currently one of the main modes of HIV transmission. A recent investigation showed that gay men and other men who have sex with men accounted for an estimated 17% of new HIV infections globally (23). However, as a potential confounding factor, sexual orientation has not been specifically considered when investigating the effects of HIV infection on brain alteration.

Considering this background, we designed a prospective MRI study, which controls for the sexual orientation in addition to other confounding factors controlled in previous HIV neuroimaging studies, to investigate the structural and functional changes in the HIV+ individuals. To be more specific, we limited our samples to HIV+ or HIV– HoM. We collected their sMRI and rs-fMRI data, performed voxel-based morphometry (VBM) and seed-based functional connectivity analysis, and calculated the ALFF and regional homogeneity values. The purpose of the current study was to determine how HIV alters the brain structure and function when sexual orientation was explicitly controlled as the homosexual males.

## Materials and Methods

### Participants

A total of 43 HIV+ HoM (age 28.23 ± 3.80 ranging from 21 to 35 years) and 40 HIV– HoM (age 27.80 ± 4.49 ranging from 19 to 35 years) were recruited in this current study. There was no significant group difference in the mean age [*t*_(81)_ = −0.475, *P* = 0.636, BF_+0_ = 0.25, with median posterior δ = −0.09, 95% CI = (−0.50, 0.31)]. The inclusion criteria were: (1) adult men aged between 18 and 35 years; (2) right-handed subjects; (3) individuals that were able to sign informed consent. The exclusion criteria were: (1) individuals with the history of confounding neurological diseases including multiple sclerosis, Parkinson's disease, epilepsy, or dementia; (2) individuals with current or past opportunistic central nervous system infection; (3) people with head injury with loss of consciousness longer than 30 min; (4) individuals with the existence of psychiatric disorders including schizophrenia, depression or anxiety; (5) individuals having a history of alcohol or drug abuse; (6) individuals with MRI contraindication. The clinical assessment characteristics are presented in Table 1. Among the 43 HIV+ HoM, 28 received the standard neurocognitive tests before the MRI scan. None of the tested participants were diagnosed as cognitive impairment (global deficit score ≥ 0.5) (24). This study was approved by the institutional review board of Shanghai Public Health Clinical Center, and all the participants provided written informed consents.

**Table 1:** Demographics, clinical information, and neurocognitive performance of HIV+ HoM and HIV– HoM.

**Category**	**HIV+ HoM** **(***n*** = 43)**	**HIV– HoM** **(***n*** =40)**	***P*** **-value**	**BF**
Age (years)	28.23 ± 3.80	27.80 ± 4.49	0.636	0.25
Duration of HIV infection (months)	43.00 (18.00 − 66.00)	†		
Duration of cART (months)	39.00 (17.00 − 57.00)	†		
Current CD4+ cell count (cells/μl)	479.65 ± 179.56 (353.00 − 640.00)	†		
Nadir CD4+ cell count (cells/μl)	247.86 ± 117.76 (183.00 − 310.00)	†		
Current CD8+ cell count (cells/μl)	807.47 ± 282.41 (622.00 − 994.00)	†		
Highest CD8+ cell count (cells/μl)	1,140.23 ± 451.26 (819.00 − 1,381.00)	†		
Current viral load		†		
<20, *n* (%)	9 (21%)	†		
Not detected, *n* (%)	31 (72%)	†		
GDS, *n*	0.27 ± 0.29 (0.07 − 0.36), 28	†		

*Data were shown in mean ± SD; IQR, interquartile range; extremum*.

*^†^, Not measured; and P-values were computed based on two-sample t-test. BF, Bayes factor; GDS, global deficit score*.

### MRI Data Acquisition

Scanning was performed using an Ingenia 3.0 T scanner (Philips, Amsterdam, Netherlands) with an 8-channel phase-array head coil at the Shanghai Public Health Clinical Center. For the resting-state scan, subjects were instructed to stay awake and to remain as still as possible while keeping their eyes closed. Resting-state fMRI images were acquired via an echo-planar imaging sequence (echo time = 25 ms, repetition time = 2,000 ms, flip angle = 75°, field of view = 224 × 224 mm, axial slices = 34, slice thickness = 4 mm, and voxel size = 1.0 × 1.0 × 1.0 mm, 200 time points). T1-weighted structural images were obtained on a sagittal orientation employing a magnetization-prepared rapid gradient-echo sequence (echo time = 3.8 ms, repetition time = 1,900 ms, flip angle = 8°, field of view = 240 × 240 mm, 170 slices per slab, voxel size = 1.0 × 1.0 × 1.0 mm, and matrix = 256 × 256).

### MRI Data Analysis

The VBM analysis was performed using Statistical Parametric Mapping (SPM12) with the VBM8 toolbox. The raw data were first checked for the scanner artifacts and anatomical abnormalities. Subsequently, the whole-brain T1-weighted images were segmented into gray matter, white matter, and cerebrospinal fluid images, and were normalized to adjust for differences in volume. Finally, all segmented images were smoothened with an 8 mm full width at a half-maximum (FWHM) Gaussian kernel standard to increase the signal-to-noise ratio. Significance was identified using a voxel-level threshold of *P* < 0.001 and a cluster-level threshold of *P* < 0.05 with family wise-error (FWE) correction for multiple comparisons.

The resting-state fMRI data were preprocessed using the Statistical Parametric Mapping (SPM12) and the Resting-State fMRI Data Analysis Toolkit (REST, http://www.restfmri.net). After discarding the first 10 volumes, the remaining fMRI images were realigned, and slice-timing was corrected. The images were then co-registered to individual T1-weighted images and spatially normalized using the Montréal Neurological Institute (MNI) template. We removed subjects who had head motion exceeding 3.0 mm of maximal translation (in any direction of x, y, or z) or 3.0° of maximal rotation during the course of scanning. The normalized images were re-sampled to an isotropic voxel size of 3.0 × 3.0 × 3.0 mm and smoothened using an FWHM Gaussian kernel of 6 mm before they were subjected to the removal of the linear drift. Six head motion parameters, and ventricle and white matter signals were regressed out before temporal filtering (0.01–0.08 Hz).

The clusters identified from the VBM analysis were defined as regions of interest (ROIs) and served as the seeds in the seed-based functional connectivity analysis. For each participant, the time courses of voxels in each ROI were extracted and averaged across voxels. Pearson correlation was computed between the seed time series and time series of other voxels in the brain, and the correlation coefficients were transformed into Fisher's Z-scores. Group differences were compared with controlling age as a covariate. Statistical significance was identified using a voxel-level threshold of *P* < 0.001 and a cluster-level threshold of *P* < 0.05 with FWE correction for multiple comparisons.

The ALFF value of each voxel was calculated by averaging the square root of the power spectrum with 0.01–0.08 Hz. The regional homogeneity value of each voxel was estimated by calculating Kendall's coefficient of concordance of the given voxel along with its adjacent 26 voxels. Group comparisons of ALFF and regional homogeneity values were performed using the two-sample *t*-test with age controlled as a covariate, and significance was identified using a voxel-level threshold of *P* < 0.001 and a cluster-level threshold of *P* < 0.05 with FWE correction for multiple comparisons.

In addition to the standard framework of frequentist statistics featuring *P*-value null-hypothesis significance testing, we calculated the Bayes factors (BF_+0_) based on *t*-values and sample sizes using the BayesFactor package in R (https://cran.r-project.org/web/packages/BayesFactor/index.html) (25). BF_+0_ ≥ 3 provides evidence for the alternative hypothesis; BF_+0_ ≤ 1/3 provides evidence for the null hypothesis; 1/3 ≤ BF_+0_ ≤ 3 indicate that there is insufficient evidence to draw a conclusion for or against either hypothesis (26).

## Results

**Figure 1:**
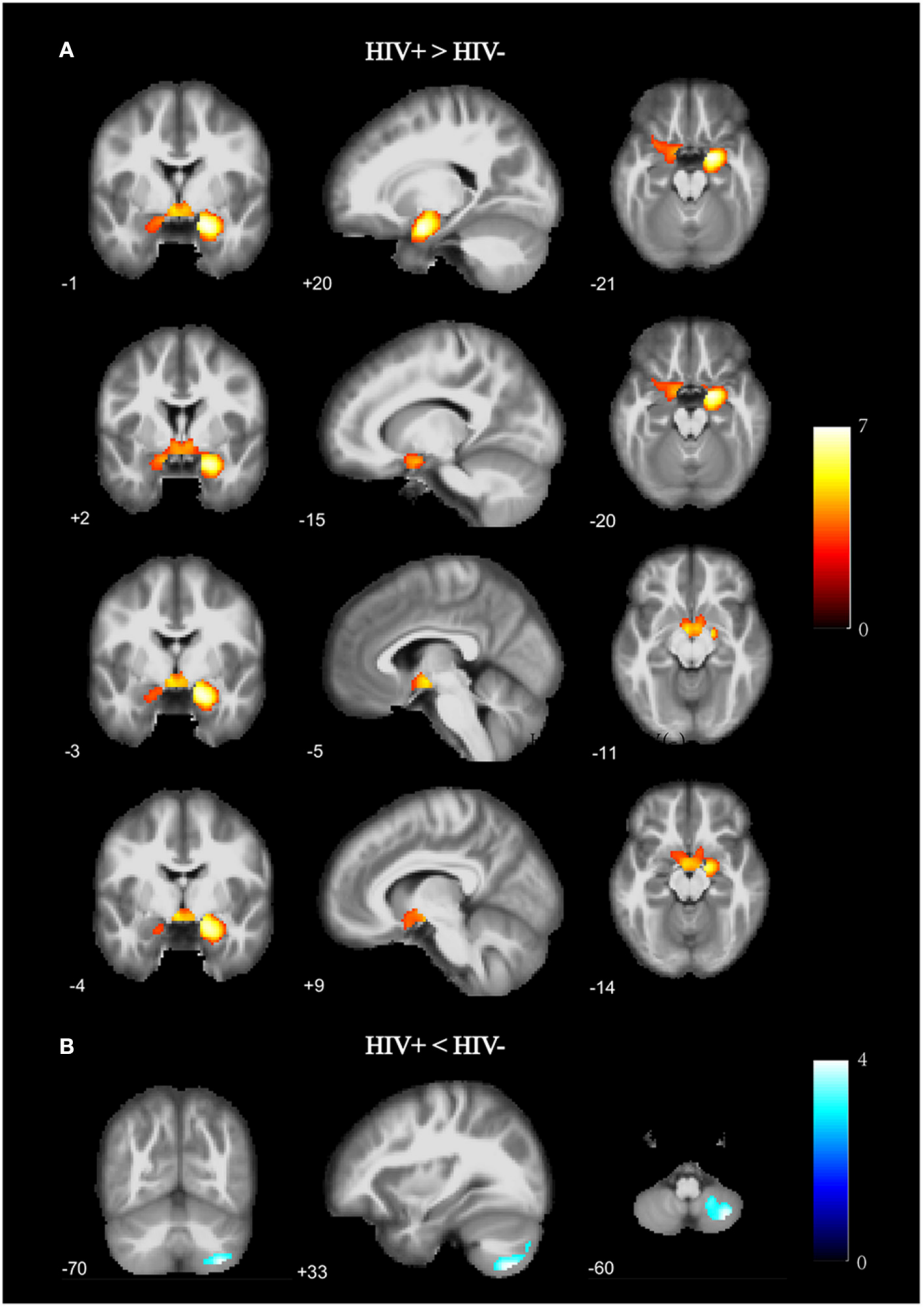
Gray matter volume (GMV) differences between HIV+ HoM and HIV– HoM based on voxel-based morphometry (VBM) analysis. **(A)** Brain regions with significantly increased gray matter in the HIV+ HoM group (voxel-level uncorrected *P* < 0.001, cluster-level FWE-corrected *P* < 0.05). **(B)** Brain regions with significantly decreased gray matter in the HIV+ HoM group (voxel-level uncorrected *P* < 0.001, cluster-level FWE-corrected *P* < 0.05). The color bars indicate *T*-statistics.

As shown in Figure 1 and Table 2, the HIV+ HoM group had significantly higher GMV mainly in the bilateral parahippocampal gyrus (ParaHippocampal_R and ParaHippocampal_L, brain labels were based on the AAL3 atlas) and amygdala (Amygdala_L and Amygdala_R) compared with HIV– HoM controls after controlling for age but reduced GMV were observed in the right inferior cerebellum (Cerebelum_8_R) (FWE corrected, voxel-level *P* < 0.001, cluster-level *P* < 0.05).

**Table 2:** Gray matter volume differences between HIV+ HoM and HIV– HoM.

**Region**	**Coordinates**			**Cluster Size**	***T*** **-value**	***P*** **-value**	**BF**
	**x**	**y**	**z**				
HIV+ > HIV–							
** ParaHippocampal_R**	20	−1	−21	3,614	7.281	<0.0000	39,130,564
** Amygdala_L**	−5	−3	−11	3,614	5.293	<0.0000	13,729
** Amygdala_R**	9	−4	−14	3,614	4.659	<0.0000	1,429
** ParaHippocampal_L**	−15	2	−20	3,614	4.613	<0.0000	1,220
Cerebelum_8_L	−17	−69	−38	95	4.760	<0.0000	2,024
Putamen_L	−30	−9	6	62	4.375	<0.0000	550
HIV+ < HIV–							
** Cerebelum_8_R**	33	−70	−60	874	−4.296	0.0001	424
Calcarine_L	0	−78	7	715	−4.121	<0.0000	242
Cerebelum_Crus2_L	−32	−83	−41	337	−4.506	<0.0000	849
Hippocampus_R	27	−27	−5	206	−5.065	<0.0000	5,969
Thalamus_R	12	−29	12	145	−4.061	0.0001	201
Cerebelum_6_R	8	−65	−15	67	−3.548	0.0007	44

*BF, Bayes factor. Brain regions at a voxel-level threshold of P < 0.001 and a cluster-level threshold of P < 0.05 with FWE correction for multiple comparisons were highlighted*.

**Figure 2:**
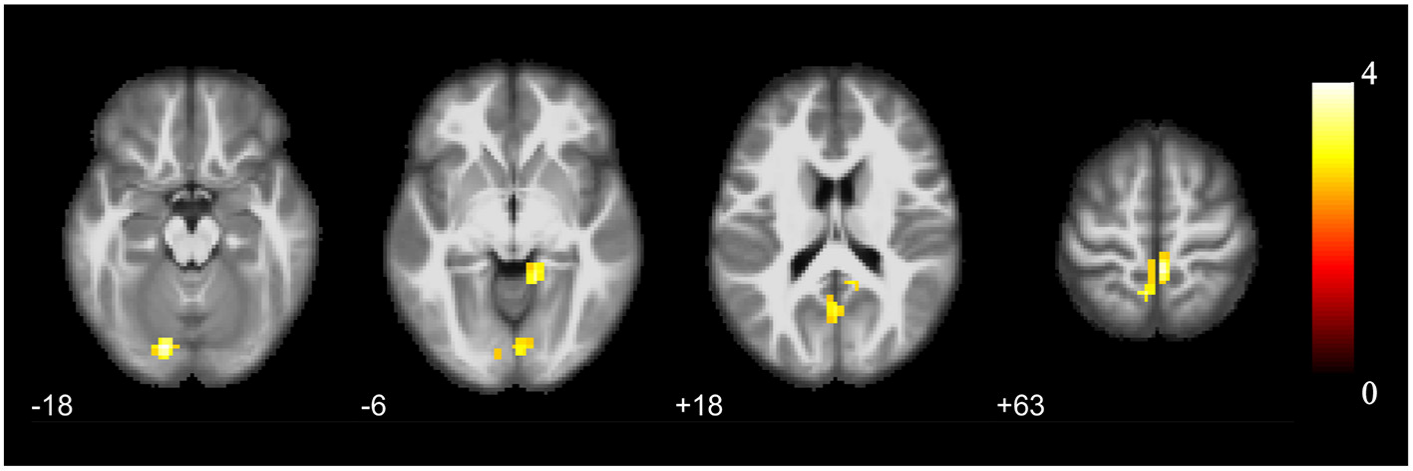
Brain regions with increased functional connectivity to the cluster with increased GMV (parahippocampal gyrus and amygdala) in HIV+ HoM compared with HIV– HoM (voxel-level uncorrected *P* < 0.005, cluster size > 50). The color bars indicate *T*-statistics.

The cluster with increased GMV in the parahippocampal gyrus and amygdala found in the VBM analysis was then used as the seed in the seed-based functional connectivity analysis of rs-fMRI data. No significant group difference was found using the strict threshold (voxel-level *P* < 0.001, cluster-level *P* < 0.05 with FWE correction). With a less strict threshold (voxel-level uncorrected *P* < 0.005, cluster size > 50), the functional connectivity strength of the cluster with the left superior cerebellum (Cerebelum_Crus1_L), right lingual gyrus (Lingual_R), and left precuneus (Precuneus_L) was decreased in the HIV+ HoM group compared with the HIV– HoM controls as shown in Figure 2 and Table 3.

**Table 3:** Functional connectivity differences between HIV+ HoM and HIV– HoM.

**Region**	**Coordinates**			**Cluster Size**	***T*** **-value**	***P*** **-value**	**BF**
	**x**	**y**	**z**				
HIV+ > HIV–							
Cerebelum_Crus1_L	−15	−84	−18	96	4.022	0.0001	178
Lingual_R	12	−45	−6	92	3.619	0.0005	53
Precuneus_L	0	−63	18	57	3.198	0.0020	17

*BF, Bayes factor. Corrected for multiple comparisons (voxel-level uncorrected P < 0. 005, cluster size > 50)*.

**Figure 3:**
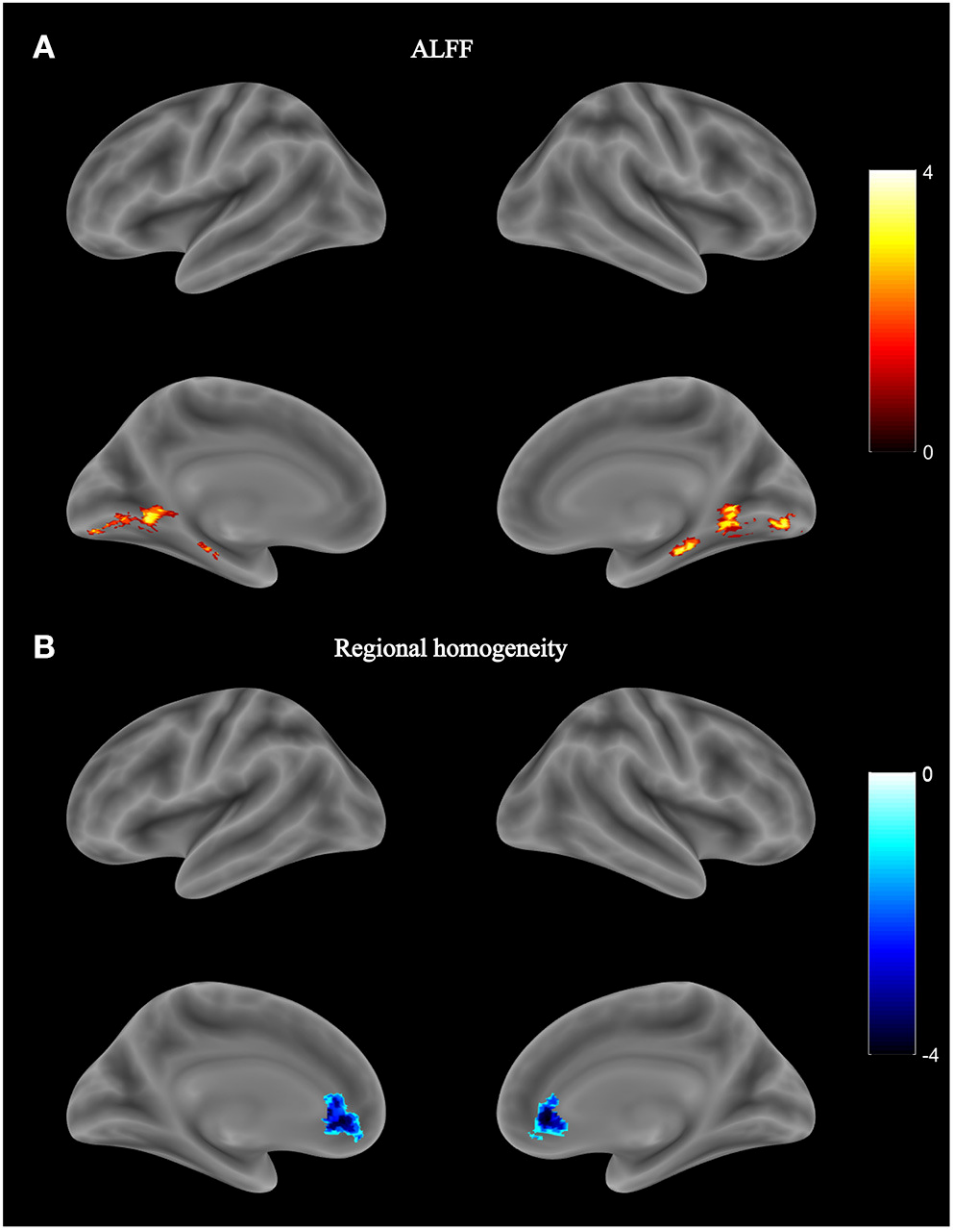
Local functional abnormality in HIV+ HoM compared with HIV– HoM. **(A)** Brain regions with increased amplitude of low-frequency fluctuation (ALFF) values in the HIV+ HoM group (voxel-level uncorrected *P* < 0.005, cluster-level FWE-corrected *P* < 0.05). **(B)** Brain regions with decreased regional homogeneity in the HIV+ HoM group (voxel-level uncorrected *P* < 0.005, cluster size > 50). The color bars indicate *T*-statistics.

No significant group difference was found in ALFF and regional homogeneity values using the strict threshold (voxel-level *P* < 0.001, cluster-level *P* < 0.05 with FWE correction). With a less strict threshold (voxel-level uncorrected *P* < 0.005, cluster-level *P* < 0.05), the HIV+ HoM exhibited an increase in ALFF in the right fusiform gyrus (Fusiform_R) and left parahippocampal gyrus (ParaHippocampal_L) as shown in Figure 3A and Table 4. With a less strict threshold (voxel-level uncorrected *P* < 0.005, cluster size > 50), the HIV+ HoM exhibited a decrease in regional homogeneity in the right anterior cingulate and paracingulate gyri (Cingulate_Ant_R), and left superior cerebellum (Cerebelum_Crus1_L) as shown in Figure 3B and Table 5.

**Table 4:** Amplitude of low-frequency fluctuation (ALFF) differences between HIV+ HoM and HIV– HoM.

**Region**	**Coordinates**			**Cluster Size**	***T*** **-value**	***P*** **-value**	**BF**
	**x**	**y**	**z**				
HIV+ > HIV–							
Fusiform_R	24	−33	−21	147	3.814	0.0003	94
ParaHippocampal_L	−21	−27	−18	210	3.778	0.0003	85

*BF, Bayes factor. Corrected for multiple comparisons (voxel-level uncorrected P < 0. 005, cluster-level FWE-corrected P < 0.05)*.

**Table 5:** Regional homogeneity differences between HIV+ HoM and HIV– HoM.

**Region**	**Coordinates**			**Cluster Size**	***T*** **-value**	***P*** **-value**	**BF**
	**x**	**y**	**z**				
HIV+ < HIV–							
Cingulate_Ant_R	3	33	0	195	−4.328	0.0000	471
Cerebelum_Crus1_L	−18	−72	−30	56	−3.678	0.0004	63

*BF, Bayes factor. Corrected for multiple comparisons (voxel-level uncorrected P < 0.005, cluster size > 50)*.

## Discussion

The current study investigated the impact of HIV on brain structure and function in a cohort of adults with the same sexual orientation. We found that, in homosexual males, HIV infection showed greater GMV of limbic structures (parahippocampal gyrus and amygdala), and increased functional connectivity of these limbic structures with cerebellum, and brain regions associated with the visual network (middle occipital gyrus and lingual gyrus). These results provided evidence of brain abnormalities in people living with HIV infection.

Our major finding was the structural and functional dysfunction of the parahippocampal gyrus in the HIV+ HoM. Several previous studies have repeatedly reported the impact of HIV infection on the parahippocampal gyrus. A previous study comparing the HIV+ older adults with age-matched uninfected controls showed reduced GMV in the parahippocampal gyrus (27). Another study about people living with HIV infection also found brain atrophy in the hippocampus/parahippocampal gyrus (28). In contrast to these previous findings, our main finding was that the HIV+ HoM group had a greater GMV in the bilateral parahippocampal gyrus compared with controls. Compared to the relative abundance of literature elucidating the structural atrophy to inflammation and immune function (29–31), the mechanisms of pathogenesis, such as hypertrophy of brain structures have not been well-understood. As we studied a relatively young cohort compared to previous studies, we speculated that the increased GMV of the parahippocampal gyrus may have been related to the early compensation of brain functions due to the brain parenchymal lesions induced by HIV infection. This speculation was further supported by the enhanced regional function in this region as indicated by the increased ALFF. The parahippocampal gyrus has been associated with many cognitive functions, especially in episodic memory and visuospatial processing (32). Our study also reported increased functional connectivity between the parahippocampal gyrus and the precuneus, which participates in memory-related activities and is closely connected to the hippocampus (33). Thus, the abnormalities we reported in the parahippocampal gyrus may affect memory, providing supporting evidence to the memory dysfunction associated with HIV infection (34, 35).

In addition to the parahippocampal gyrus, the amygdala also showed increased GMV in this study. As an important nucleus that can affect mood, emotion, learning, and memory function, the GMV changes in the amygdala were often involved in neurological or psychiatric diseases, such as Asperger syndrome (36), anxiety (37), and advanced depression (38). A neuroimaging study involving HIV+ individuals exposed to early life stress showed that high levels of stress and HIV could interact to increase the amygdala volume and may result in neurocognitive dysfunction in HIV+ individuals (39). In terms of sexual orientation, the amygdala is an important component of the core neural pathway of male sexual arousal and is responsible for emotional response regulation (40). The activation of the amygdala in HoM was greater than heterosexual males in response to specific visual stimuli (41). It is possible that the sexual orientation and HIV could also interact to increase the amygdala volume. However, the potential interaction between male homosexual orientation and HIV infection remains to be further investigated. In addition, the cingulate gyrus, which is reciprocally connected with amygdala, showed decreased regional homogeneity in the HIV+ HoM of our study.

We found decreased GMV in the cerebellum in the HIV+ HoM, which is consistent with previous studies (42, 43). The function of left superior cerebellum was also disrupted as indicated by the decreased regional homogeneity in the HIV+ HoM. Furthermore, we found increased functional connectivity between the left superior cerebellum and the cortical areas with greater GMV (mainly the parahippocampal gyrus) in the HIV+ HoM group comparing to the HIV– HoM. This is in accordance with the previous studies indicating important functional interactions between the cerebellum and the hippocampus formation. For instance, functional connectivity between the left hippocampus and the bilateral cerebellum was increased in HIV+ individuals, which was associated with the spatio-temporal prediction of movements in the memory formation (44). In a study of cerebellar functional connectivity in HIV+ male individuals, decreased functional connectivity between the right lobule VI and the left hippocampus was considered as the brain mechanism underlying the impairment of spatial and temporal processing function (45). As the parahippocampal gyrus is the main surrounding structure of the hippocampus and plays an important role in memory encoding and retrieval (46), the increased functional connectivity between the parahippocampal gyrus and cerebellum found in the current study might similarly indicate the behavioral impairments in memory functions.

The HIV+ HoM group also exhibited functional abnormality in the brain regions involved in the visual network. The lingual gyrus plays an important role in visual attention and visual judgment (46). As discussed previously (41), the HoM showed greater activation of the amygdala when processing specific visual stimuli. The increased functional connectivity we found between the visual areas and brain structures with increased GMV including the amygdala in the HIV+ HoM group might suggest a certain relationship between the HIV infection and HoM. We also found a local functional abnormality in the visual network in the HIV+ HoM, demonstrated by the increased ALFF values in the fusiform gyrus involved in visual processing. As little evidence has been reported in previous studies, the relationship between visual cortex dysfunction and HIV infection needs to be further studied.

There were several limitations in this study. First, the grouping of sexual orientation was based on the self-identification of participants, lacking a measurement basis such as the Kinsey scale (47). We might have included participants who were “homosexual but with incidental or occasional heterosexual tendencies,” which is separated from “exclusive homosexual” on the Kinsey scale. The current findings need to be further verified using exclusive homosexual individuals. Second, the clinical information of enrolled individuals was incomplete, including some important confounding factors, such as the medication regimen of HIV+ patients. And although individuals having a history of alcohol or drug abuse were excluded in our study, these conditions were obtained via self-report and concealment of illicit drug use could not be ruled out. Thus, we could not completely eliminate the influence of such confounding factors on the results. Third, the present study did not conduct a full set of neurocognitive tests in all HIV+ patients, and the explanation for the positive results of structural and functional MRI was mainly dependent on the reports from other scholars. Future research involving a specific neurocognitive performance may help address some of these questions.

To our best knowledge, this is the first study to strictly control sexual orientation while investigating the HIV-related alteration in the brain structure and function. We found significant structural abnormalities of the limbic system and cerebellum, and changes in functional connectivity and activity in the brain regions related to memory and visual function in the HIV+ HoM compared with the HIV– HoM controls. Further studies are needed to expand the sample size, acquire relevant clinical information, and group strictly according to confounding factors, and employ multimodal imaging methods, so as to improve our understanding of the pathophysiological mechanism of HIV infection in the brain.

## Data Availability Statement

The original contributions presented in the study are included in the article/supplementary material, further inquiries can be directed to the corresponding author/s.

## Ethics Statement

The studies involving human participants were reviewed and approved by the Ethics Committee of the Shanghai Public Health Clinical Center. The patients/participants provided their written informed consent to participate in this study.

## Author Contributions

QM and XS: study design, participants recruitment, data collection, and data analysis. GC: data collection and data analysis. FS and FL: data analysis. HZ: participants recruitment. YS and D-CC: study conceptualization, project administration, and data analysis. All authors contributed to the article and approved the submitted version.

## Funding

This work was supported by the Foundations of Shanghai Municipal Population and Family Planning Commission (201840146) and Science and Technology Commission of Shanghai Municipality (19411965800).

## Conflict of Interest

The authors declare that the research was conducted in the absence of any commercial or financial relationships that could be construed as a potential conflict of interest.

## Publisher's Note

All claims expressed in this article are solely those of the authors and do not necessarily represent those of their affiliated organizations, or those of the publisher, the editors and the reviewers. Any product that may be evaluated in this article, or claim that may be made by its manufacturer, is not guaranteed or endorsed by the publisher.
